# An Innovative Semiparametric Density Model for the Statistical Characterization of Ground-Vehicle Radar Cross Sections

**DOI:** 10.3390/s26092572

**Published:** 2026-04-22

**Authors:** Zengcan Liu, Shuhao Wen, Houjun Sun, Ming Deng

**Affiliations:** 1 School of Integrated Circuits and Electronics, Beijing Institute of Technology, Beijing 100081, China; 3120195368@bit.edu.cn (Z.L.); sunhoujun@bit.edu.cn (H.S.); 2 Southwest Technology and Engineering Research Institute, Chongqing 401329, China; 3 Key Laboratory of Optoelectronic Technology & Systems (Ministry of Education), Chongqing University, Chongqing 400044, China; wen2228004545@outlook.com

**Keywords:** statistical characterization, radar cross section, semiparametric density estimation, kernel density estimation

## Abstract

Accurately characterizing the statistical fluctuations of vehicle radar cross sections (RCSs) across polarization states and azimuthal sectors is essential for evaluating detection performance, conducting probabilistic simulations, and analyzing target features in millimeter-wave radar systems. Existing one-dimensional RCS statistical models, including Weibull, Chi-square, Lognormal, Rice, and Gaussian distributions, are often limited by their restricted functional expressiveness, making it difficult to simultaneously capture skewness, tail thickness, and azimuthal dependence under narrow angular-domain conditions. In addition, purely nonparametric approaches tend to produce spurious modes under finite-sample conditions and lack interpretable structural priors. To address these limitations, this paper proposes a Unimodal RCS Semiparametric Density Estimator (URCS-SDE) tailored for ground-vehicle targets. The proposed approach adopts kernel density estimation (KDE) as a data-driven baseline representation and incorporates physically plausible structural constraints through unimodal shape projection. Then a beta-type tail template is further introduced in the normalized amplitude domain to regulate boundary decay behavior. Finally, weighted least-squares calibration is performed on the histogram grid of the empirical probability density function (PDF), achieving a balanced trade-off between fitting accuracy and stability in both the peak and tail regions. Using multi-azimuth RCS measurements of two representative ground vehicles, the URCS-SDE is systematically compared with five classical parametric distributions and a representative regularized mixture density network (MDN) baseline. Performance is evaluated under both full-azimuth and directional-window conditions using the sum of squared errors (SSE), root mean squared error (RMSE), coefficient of determination (R-square) and held-out negative log-likelihood (NLL). The results show that the URCS-SDE consistently provides the most accurate and stable density estimates, especially in narrow angular windows. In addition, a threshold-based detection-support example derived from the fitted PDFs demonstrates that the advantage of the URCS-SDE transfers from density reconstruction to a directly engineering-relevant downstream quantity.

## 1. Introduction

Radar cross section (RCS) is one of the fundamental physical quantities characterizing a target’s ability to reflect incident electromagnetic waves, and it directly influences radar detection range, tracking continuity, and false-alarm and missed-detection probabilities, as well as target recognition performance [1,2,3,4]. For ground vehicles, the statistical fluctuation characteristics of RCSs are not only critical to surveillance applications involving maneuvering ground targets but are also closely tied to safety perception in automotive radar systems for autonomous driving and advanced driver-assistance systems [5]. In recent years, safety-oriented virtual testing and digital twin technologies have increasingly become essential pathways in the development of automotive perception systems, where the credibility of radar sensor models largely depends on the statistical characterization of realistic scattering fluctuations. Therefore, reliance solely on mean RCS values or a limited number of empirical samples is insufficient to support system-level Monte Carlo simulations and robustness assessments [6]. Moreover, performance evaluation and virtual testing commonly rely on statistical assumptions of echo fluctuations when estimating detection probability, false-alarm probability, and detection thresholds. And if the shape of the underlying probability density function (PDF) is inaccurately characterized, it can directly lead to biased detection-range estimation and inconsistencies in statistical simulations [7]. Therefore, rather than reporting only mean RCS values or a small set of statistical descriptors, establishing PDF models that remain stable across multiple polarizations and azimuthal conditions, including narrow angular-domain windows, is a necessary prerequisite for reliably integrating measured scattering behavior into system-level evaluation chains.

Conventional statistical modeling of RCSs and clutter is commonly associated with the Swerling I–V target fluctuation models and classical one-dimensional amplitude distributions [8]. Distributions such as Chi-square, Lognormal, Weibull, Rice, and Gaussian have been widely employed to characterize the fluctuation behavior of targets and clutter across different frequency bands and environmental conditions [9,10]. Owing to their simple functional forms and strong analytical tractability, these models are well suited to scenarios in which target fluctuations are relatively mild or the scattering mechanisms are comparatively simple. However, numerous studies have shown that when RCS distributions exhibit pronounced skewness, heavy tails, or even multimodal structures, a single classical distribution often provides limited fitting accuracy and struggles to comprehensively characterize the statistical behavior of complex aircraft or vehicle targets under maneuvering and dynamic conditions [11,12,13,14].

To improve the accuracy of dynamic RCS modeling, a variety of more flexible parametric and composite models have been proposed in recent years. For aircraft targets, Wang et al. proposed an innovative bimodal RCS statistical model that represents the bimodal behavior of dynamic aircraft RCSs by combining two parametric distributions. Their results indicate that, when the RCS PDF exhibits a pronounced bimodal pattern, the proposed model can significantly outperform single-distribution baselines such as Weibull, Lognormal, and Chi-square. However, this approach inherently assumes that the underlying distribution is bimodal, and its parameter tuning can be relatively complex [15]. Hughes introduced a piecewise cumulative Weibull model, which approximates complex RCS distributions by stitching multiple Weibull components over different intervals. Although this strategy increases modeling flexibility, it also leads to a more complex structure and reduced interpretability of the parameters [16]. In addition, composite models formed by combining distributions such as Chi-square and Lognormal have been explored for fitting dynamic RCSs. While effective in certain scenarios, these models often introduce a larger number of parameters and may suffer from limited generalization capability [17]. For ground targets and automotive scenarios, existing studies have largely focused on measurement campaigns and relatively simple statistical modeling. Raynal et al. conducted a statistical analysis of Ku-band RCS measurements for representative ground vehicles and highlighted the importance of RCS statistics in detection algorithm design; however, their characterization primarily relied on classical one-dimensional distribution models [18]. In the millimeter-wave automotive radar band (e.g., 77 GHz), Lee et al. measured the RCSs of vehicles and human targets using frequency-modulated continuous-wave radar and proposed a “pseudo-RCS” metric for automotive systems. Nevertheless, the statistical modeling of the RCS distribution remained relatively coarse [19]. Deep et al. investigated pedestrian RCS characteristics in the automotive radar band through electromagnetic simulations, with an emphasis on geometric modeling and simulation fidelity, while placing comparatively less focus on the probabilistic distribution model itself [20]. Magosi et al. proposed a semi-physical automotive radar sensor modeling approach based on experimental data for the virtual validation of autonomous driving functions. Their work further underscored the importance of realistic RCS behavior for simulation credibility yet did not provide a dedicated PDF modeling scheme specifically tailored to ground vehicles [21]. Overall, these studies have enriched the empirical understanding of ground-target RCSs. However, most of them still rely on simple parametric distributions or histogram-based statistics, and there remains a lack of a dedicated density model that simultaneously offers shape constraints, high fitting accuracy, and engineering interpretability.

In parallel with advances in RCS applications, the statistical community has made substantial progress in nonparametric and semiparametric density estimation. Kernel density estimation (KDE), as a representative nonparametric approach, can characterize skewed and multimodal distributions without prespecifying a functional form. Its theoretical properties and bandwidth selection have been systematically investigated in fields such as econometrics [22,23,24]. However, under finite-sample conditions, KDE is prone to generating spurious modes in low-density regions and has difficulty naturally enforcing structural priors such as unimodality and bounded support. To address these issues, Hjort and Glad proposed nonparametric density estimation with a parametric start, in which a baseline parametric model is multiplied by a nonparametric correction factor to enhance modeling flexibility [25]. Subsequent studies further introduced locally parametric nonparametric density estimation, combining a parametric structure with local kernel smoothing [26]. These developments indicate that semiparametric strategies integrating physical priors with data-driven modeling possess inherent advantages for problems such as RCS characterization, where prior knowledge exists alongside the need for high-accuracy fitting. On the other hand, deep learning models have been widely applied to general density estimation problems in recent years [27,28,29]. Mixture density networks (MDNs) approximate complex multimodal distributions by using neural networks to output the weights, means, and variances of Gaussian mixtures [30,31]. At the same time, normalizing flows learn invertible transformations that map simple base distributions to complex target distributions [32,33]. However, in the one-dimensional, azimuth-resolved RCS modeling setting considered in this work, deep generative models typically require substantially larger datasets and more elaborate training and regularization strategies to avoid overfitting. Moreover, their parameters’ interpretability and deployment cost do not necessarily align with engineering requirements for explicitly controllable shape priors. Therefore, this work adopts a semiparametric framework that retains data-driven flexibility while explicitly introducing unimodality and tail constraints, achieving a practical balance between modeling accuracy and engineering usability.

In summary, existing statistical modeling of ground-vehicle RCSs still lacks a one-dimensional PDF model that simultaneously satisfies the following engineering requirements: (1) robustness in narrow angular domains: maintaining smoothness and stability as the azimuthal window shrinks and sample size decreases, while avoiding KDE-induced spurious modes; (2) injectable shape priors: explicitly enforcing unimodality and providing controllable constraints on boundary and tail decay to comply with physical scattering principles; (3) accuracy–complexity trade-off: offering lower training costs and stronger interpretability than deep generative density models, thereby facilitating engineering deployment; (4) strict multi-condition validation: enabling systematic and reproducible experimental comparisons across multiple elevations, polarizations, and both full-azimuth and directional-window scenarios, evaluated using multiple performance metrics.

**Figure 1:**
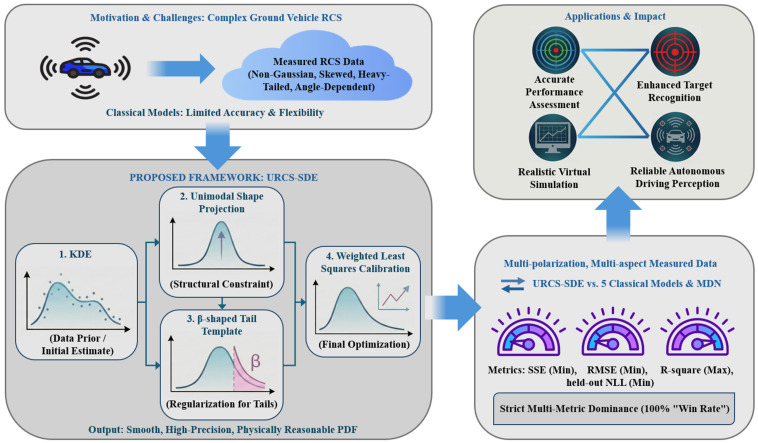
A schematic overview of the research framework, from the identification of limitations in current RCS modeling to the development and rigorous validation of the innovative URCS-SDE, highlighting its superior performance and practical applications in radar system design.

To address these challenges, this paper proposes a Unimodal RCS Semiparametric Density Estimator (URCS-SDE) for statistical characterization of ground-vehicle RCSs and makes the following contributions: (1) A structurally constrained semiparametric density framework: a one-dimensional semiparametric PDF modeling pipeline is developed by combining a data-adaptive core, explicit shape-prior constraints, and tail-template regularization. Specifically, KDE captures the overall skewed or heavy-tailed structure, unimodal projection suppresses spurious oscillations, and a beta-type tail template enforces physically consistent bounded support and boundary decay. (2) Robust calibration for narrow angular domains: a weighted least-squares calibration strategy is designed to adaptively increase weights in the peak and tail regions, thereby preserving peak alignment and tail fidelity even when the number of samples in directional windows is reduced. (3) Strict multi-condition validation using four metrics: based on two vehicle categories, multiple elevations, and polarization channels, systematic comparisons are conducted against five classical parametric distributions and a representative modern probabilistic baseline (a regularized MDN) under both full-azimuth and directional-window scenarios, thereby enabling a clearer assessment of the incremental value of the proposed semiparametric design. A unified criterion of simultaneous superiority across all four metrics is adopted to ensure reproducible conclusions independent of single-metric bias. (4) Engineering usability analysis: the engineering relevance of the fitted densities is demonstrated through a simple threshold-based detection-support application and provides implementation-level reproducibility support via scenario-accounting, default-constant, and sensitivity tables in the Supplementary Materials. The overall workflow of the proposed approach is illustrated in Figure 1. The remainder of this paper is organized as follows. Section 2 presents the classical distribution baselines and the URCS-SDE modeling procedure. Section 3 describes the experimental data, angular-domain configurations, and evaluation metrics. Section 4 reports systematic comparison results under both full-azimuth and directional-window scenarios, discusses their engineering implications and provides a simple application example based on threshold-exceedance probability. Section 5 concludes the paper and outlines future work.

## 2. Methodology

### 2.1. Classical Parametric RCS Models and Modern Probabilistic Models

To establish representative baselines, five widely used distributions in radar RCS and clutter modeling are considered: the Chi-square, Lognormal, Weibull, Rice, and Gaussian distributions. These models have been extensively studied in both the statistical and radar literature, and their standard formulations and physical interpretations are well documented; therefore, they are not reiterated here [34,35,36,37].

For each azimuthal scenario and channel, only the raw RCS amplitude samples are used, and model parameters are estimated via maximum likelihood estimation (MLE), without introducing any additional smoothing, shape constraints, or postprocessing. The resulting PDFs are then evaluated on a common amplitude grid to facilitate subsequent comparisons. This setup ensures that the classical models are assessed in their canonical forms, thereby providing an unbiased baseline for evaluating the effectiveness of the proposed URCS-SDE framework.

In addition to the five classical parametric models, the paper includes a representative modern probabilistic baseline, namely a regularized MDN. The specific principle of this model can be referred to in paper [38]. An MDN is selected because it is a canonical neural conditional density model with explicit probabilistic outputs, it has already been used in automotive radar modeling and virtual simulation, and it provides a practically reproducible deep baseline for the present one-dimensional limited-sample setting [30]. To ensure fairness, the MDN is evaluated under the same scenario partition, angular-window definition, output grid, and goodness-of-fit metrics as all other models. The purpose of this additional baseline is not to maximize neural-model complexity but to provide a stronger modern comparator for assessing the incremental value of the URCS-SDE.

### 2.2. URCS-SDE

The URCS-SDE is a one-dimensional semiparametric density estimator tailored to narrow-angle RCS data. The selected basis family is not introduced by empirical convenience from an unrestricted function dictionary. Instead, it is derived from three design requirements of azimuth-resolved RCS density modeling: (1) interior adaptivity to skewed empirical shapes induced by varying scattering-center contributions; (2) suppression of finite-sample spurious modes under a weak single-regime prior appropriate for typical narrow-angle windows; (3) bounded-domain, asymmetric control of boundary decay after amplitude normalization. These three requirements motivate, respectively, a KDE basis, a unimodal projection basis, and a beta-type tail basis.

More specifically, the KDE component preserves the local data-adaptive shape and serves as an interior pilot estimator. The unimodal projection component regularizes this pilot estimate by enforcing a monotonically increasing left branch and a monotonically decreasing right branch, thereby removing oscillations that are difficult to interpret physically under limited sample sizes. The beta-type tail component is defined on the normalized interval [0, 1]; near the left boundary, it behaves as $u^{a-1}$ and near the right boundary as ${\left(1-u\right)}^{b-1}$, so the two parameters *a* and *b* independently control the boundary orders. For a>1 and b>1, the template is positive, has bounded support, and is unimodal with a single interior mode, which makes it suitable as a weak tail regularizer rather than as a rigid global parametric model.

This design also explains why alternative choices are not adopted in the present study. Gamma-type tails are naturally defined on [0, ∞) and provide one-sided unbounded decay, which does not match the bounded normalized domain used here without additional transformations. Likewise, although the Epanechnikov kernel is asymptotically optimal for unconstrained KDE in the asymptotic mean integrated squared error (AMISE) sense, the URCS-SDE does not use KDE as the final estimator; instead, KDE serves as a smooth pilot that is subsequently regularized by unimodal projection and bounded-domain tail control. The Gaussian kernel is therefore retained because its $C^{\infty }$ smoothness is numerically advantageous for projection, interpolation, and final smoothing. It is emphasized that our basis function combination is not claimed to be the only admissible basis family but rather a structurally motivated and minimally sufficient choice for the present one-dimensional narrow-angle RCS problem. In addition, unimodality here is adopted as a local weak prior for typical rigid ground vehicles under fixed elevation, co-polarization, and narrow aspect-window conditions, rather than as a universal physical law for all targets or for all local aspect manifolds.

#### 2.2.1. Basis Functions

Let the one-dimensional RCS amplitude be denoted by x∈R. On a uniform grid ${\left\{x_{j}\right\}}_{j=1}^{M}$, three types of basis functions are defined as follows:

1. KDE basis function $f_{KDE}(x)$.

Kernel density estimation with a Gaussian kernel is applied to the raw samples, yielding ${\hat{f}}_{KDE}\left(x_{j}\right)$ on the grid. This function serves as a directly data-driven baseline shape.

2. Unimodal projection basis function $f_{UNI}(x)$.

A unimodality constraint is imposed on $f_{KDE}(x)$. The left side of the mode is constrained to be monotonically increasing via isotonic regression, while the right side is constrained to be monotonically decreasing. Subsequent smoothing and normalization are applied to obtain an idealized unimodal shape with noise-induced oscillations removed.

3. Beta-tail basis function $f_{BETA}(x)$.

The amplitude is first normalized to the interval $\left[0,1\right]$:

(1) $$u=\frac{x-x_{min}}{x_{max}-x_{min}}\;,\;u\epsilon \left[0,1\right].$$

A beta-type tail template is then applied:

(2) $$f_{BETA}\left(x\right)\propto u^{a-1}{\left(1-u\right)}^{b-1},\;a>1,\;b>1.$$

Finally, numerical smoothing is performed. This basis function provides tail shapes with bounded support and controllable skewness, which is beneficial for suppressing KDE-induced noise near the boundaries.

#### 2.2.2. Weighted Least-Squares Calibration

Let ${\left\{C_{k}\right\}}_{k=1}^{K}$ denote the centers of the histogram bins, with corresponding empirical PDF values ${\hat{f}}_{k}$. A design matrix $D\in R^{K\times 3}$ is constructed by evaluating the three basis functions at these locations:

(3) $$D_{k,1}=f_{KDE}\left(C_{k}\right),\;D_{k,2}=f_{UNI}\left(C_{k}\right),\;D_{k,3}=f_{BETA}\left(C_{k}\right).$$

Let $\hat{f}={\left({\hat{f}}_{1},\cdots ,{\hat{f}}_{K}\right)}^{T}$. A diagonal weight matrix $W=diag(w_{k})$ is defined, where larger weights are assigned to $w_{k}$ near the peak region and at both ends of the amplitude range, emphasizing accurate peak alignment and tail fitting. The coefficient vector $\alpha ={\left(\alpha_{1},\alpha_{2},\alpha_{3}\right)}^{T}$ is obtained by solving a regularized weighted least-squares problem:

(4) $$\alpha =arg\underset{\alpha \geq 0}{\min}{\left\|W^{\frac{1}{2}}(D\alpha -\hat{f})\right\|}_{2}^{2}+\lambda {\left\|\alpha \right\|}_{2}^{2},$$

where λ>0 is a small ridge regularization parameter and the non-negativity constraint α≥0 is enforced by clipping negative coefficients. This yields an unnormalized URCS-SDE density estimate:

(5) $${\tilde{f}}_{URCS}\left(x\right)=\alpha_{1}f_{KDE}\left(x\right)+\alpha_{2}f_{UNI}\left(x\right)+\alpha_{3}f_{BETA}\left(x\right).$$

A second unimodal projection and smoothing operation is then applied to ${\tilde{f}}_{URCS}\left(x\right)$, followed by normalization, resulting in the final URCS-SDE density:

(6) $$f_{URCS}\left(x\right)=\frac{{\tilde{f}}_{URCS}\left(x\right)}{\int {\tilde{f}}_{URCS}\left(t\right)dt}.$$

To demonstrate the contribution of each key component in the URCS-SDE framework, we also performed an ablation study by independently removing each of the following components: KDE, unimodal constraints, Beta-tail regularization, and weighted calibration. Removing KDE significantly deteriorates the model’s smoothing performance, leading to high errors. Without unimodal constraints, the model fails to fit reasonable peak-shape distributions, introducing unrealistic multimodal behavior. The absence of Beta-tail regularization causes poor fitting in the tail regions, while removing weighted calibration results in less precise boundary and peak fitting, especially with limited samples.

#### 2.2.3. Order-Level Bias–Variance Interpretation and Finite-Sample Regime

To clarify the statistical role of the URCS-SDE, it is useful to compare its risk structure with those of classical parametric density estimators and standard KDE. Let $f_{0}$ denote the true one-dimensional RCS density in a given narrow-angle scenario. For a correctly specified parametric family ${\{f}_{\theta }:\;\theta \epsilon \Theta \}$, the integrated risk is dominated by a variance term of order $O(n^{-1})$. However, under model misspecification, the risk contains an irreducible approximation term:

(7) $$B_{par}^{2}={inf}_{\theta \epsilon \Theta }||f_{\theta }-f_{0}{\left|\right|}_{2}^{2}$$

so that, at the order level,

(8) $${MISE}_{par}=B_{par}^{2}+O(n^{-1}).$$

Thus, the apparent low variance of a rigid parametric family is meaningful only when the approximation bias remains sufficiently small.

By contrast, if $f_{0}$ is twice continuously differentiable, standard KDE theory gives

(9) $${MISE}_{KDE}=C_{1}h^{4}+\frac{nh}{C_{2}}+O(h^{4}+\frac{1}{nh})$$

where h is the bandwidth, $C_{1}$ is associated with curvature-induced smoothing bias, and $C_{1}$ is stochastic variance. Under the usual choice $h≍n^{-\frac{1}{5}}$, this yields the one-dimensional nonparametric rate $O(n^{-\frac{4}{5}})$. In narrow-angle directional windows, however, the effective sample size is reduced, so the variance term $\frac{nh}{C_{2}}$ becomes more prominent and may manifest itself as local oscillations or weak spurious modes.

The proposed URCS-SDE is designed for this intermediate regime. Its KDE component preserves the interior data-adaptive structure, while the subsequent unimodal projection and ridge-regularized weighted calibration act as two regularizing operations that primarily target finite-sample variance inflation. This effect can be formalized for the projection step as follows:

Let

(10) $$U_{m}=\{g\in R^{M}:g_{1}\leq \cdots \leq g_{m},\;g_{m}\geq \cdots \geq g_{M}\}$$

be the cone of grid functions that are nondecreasing up to index m and nonincreasing thereafter and let $P_{m}$ denote the Euclidean projection onto $U_{m}$. If the target grid density $f_{0}\in U_{m}$, then for any pilot estimate $g\in R^{M}$,

(11) $$||P_{m}g-f_{0}{\left|\right|}_{2}\leq ||g-f_{0}{\left|\right|}_{2}.$$

Since it is conditional on a correct mode index, the unimodal projection step cannot increase the $L_{2}$ estimation error. Since $U_{m}$ is a closed convex cone, $P_{m}$ is the metric projection in a Hilbert space. The result follows directly from the non-expansiveness property of Euclidean projections onto closed convex sets.

In the practical implementation, the mode index is estimated from the pilot density, so the above proof should be interpreted as the ideal fixed-mode analog explaining why shape projection reduces finite-sample roughness when the narrow-angle density is approximately unimodal. After the projection step, the weighted least-squares calibration further stabilizes the estimate by shrinking unstable coefficient combinations among the three basis functions. Accordingly, the risk of the URCS-SDE can be interpreted at the order level as

(12) $${MISE}_{URCS}\approx B_{s}^{2}+C_{1}h^{4}+{\tilde{C}}_{2}{\left(nh\right)}^{-1}+C_{3}\lambda^{2}$$

where $B_{s}^{2}$ denotes the residual structured bias associated with the unimodal/tail regularization, ${\tilde{C}}_{2}$ is an effective finite-sample variance constant after projection and shrinkage, and λ is the ridge parameter. This expression shows that the URCS-SDE preserves the nonparametric consistency envelope of KDE while shifting the practical bias–variance balance toward lower finite-sample variance and smaller approximation bias than rigid single-family parametric models.

This comparison also yields an order-level finite-sample regime boundary. If a misspecified parametric model has the risk $B_{par}^{2}+\frac{C_{par}}{n}$, while the URCS-SDE has a risk of approximately $B_{s}^{2}+\tilde{C}n^{-\frac{4}{5}}$, then when the following conditions are met, the URCS-SDE will outperform the model:

(13) $$n≳(\frac{\tilde{C}}{B_{par}^{2}-B_{s}^{2}}{)}^{\frac{5}{4}},\;\;B_{par}^{2}>B_{s}^{2}.$$

Although the constants depend on the target class and implementation details, this boundary explains why the URCS-SDE is especially suitable for bounded, skewed, approximately unimodal, and finite-sample narrow-angle RCS densities, where a single classical family is too rigid while raw KDE remains variance-sensitive.

Therefore, the URCS-SDE can be viewed as a dedicated semiparametric density-modeling framework for ground-vehicle RCSs. On the one hand, it integrates data-driven flexibility with physically meaningful structural priors through the combination of KDE, unimodal projection, and Beta-tail regularization. On the other hand, relative to modern neural density baselines, the URCS-SDE is intended to provide a lower-complexity and more explicitly controllable alternative for one-dimensional limited-sample RCS modeling. To substantiate this positioning, our experiments include a representative regularized MDN baseline under the same evaluation protocol as the five classic single-distribution models, so that the comparison between semiparametric and neural probabilistic modeling is assessed empirically rather than asserted a priori. To improve reproducibility, all key implementation parameters and sensitivity ranges of the URCS-SDE are explicitly fixed in the Supplementary Materials.

## 3. Experimental Setup

### 3.1. Datasets and Measurement Configuration

RCS data from two representative ground vehicles, denoted as vehicle A (a small-sized vehicle) and vehicle B (a medium-sized vehicle), are used in this study. For each vehicle, the raw data covers the full azimuthal range φ∈[0°,360°], with RCS amplitude samples indexed by the azimuth angle φ. Each data record consists of one azimuth angle and six channels, formed by the combination of three elevation-angle configurations and two co-polarization modes, $c\in \left\{20\;HH,\;20\;VV,\;40\;HH,\;40\;VV,\;60\;HH,\;60\;VV\right\}$, where Horizontal–Horizontal (HH) and Vertical–Vertical (VV) denote co-polarized returns, while 20°, 40° and 60° correspond to different elevation-angle configurations in the measurement geometry. The data are stored in tabular files, each containing one azimuth column and six RCS amplitude columns corresponding to the six channels. Accordingly, the calibrated RCS amplitude at azimuth φ and channel c is denoted by $\sigma_{c}(\varphi )$.

To characterize both global statistical behavior and directional variations, two evaluation scenarios are considered. The first is the full-azimuth scenario, in which samples from a given channel are aggregated over the entire range 0–360° to obtain a global PDF. The second is the directional-window scenario, where narrow angular windows of ±5° are defined around four representative azimuths, $\varphi_{0}\in \left\{0{}^{\circ},90{}^{\circ},180{}^{\circ},270{}^{\circ}\right\}$, corresponding to the front, right, rear, and left aspects. Then circular angular mapping is adopted to avoid discontinuities near 0° or 360°:

(14) $$A\left(\varphi_{0}\right)=\left\{\varphi :\left|wrap(\varphi -\varphi_{0})\right|\leq 5{}^{\circ}\right\}.$$

where wrap(·) accounts for the circular nature of the azimuth angle. The samples within each window, $\left\{\sigma_{c}\left(\varphi \right):\varphi \in A\left(\varphi_{0}\right)\right\}$, define a direction-dependent PDF $f_{c,\varphi_{0}}(x)$. For each vehicle v, channel c, and directional window centered at $\varphi_{0}$, the effective sample size is defined as

(15) $$N_{v,c,\varphi_{0}}=\left|\left\{\sigma_{c}\left(\varphi \right):\varphi \in A\left(\varphi_{0}\right)\right\}\right|.$$

In the implementation used in this study, a directional-window scenario is retained for quantitative comparison only when $N_{v,c,\varphi_{0}}\geq 40$; otherwise, the scenario is excluded from quantitative evaluation and counted in the scenario-accounting summary. This design captures characteristic scattering differences across the four principal aspects while ensuring that each window contains sufficient samples for stable estimation. In addition, histograms are used solely for internal quantitative metric computation, ensuring consistent evaluation criteria across different models.

### 3.2. Metric Calculation and Advantage Determination

To compare the fitting performance of the URCS-SDE with five classical models and a regularized MDN, four commonly used goodness-of-fit metrics are employed: sum of squared errors (SSE), root mean squared error (RMSE), coefficient of determination (R-square) and held-out negative log-likelihood (NLL). Let ${\hat{f}}_{k}$ denote the empirical PDF and $f_{k}$ the model-based PDF; the following metrics are then computed:

1. SSE:
  (16) $$SSE=\sum_{k=1}^{K}{\left({\hat{f}}_{k}-f_{k}\right)}^{2}.$$
2. RMSE:
  (17) $$RMSE=\sqrt{\frac{1}{K}\sum_{k=1}^{K}{\left({\hat{f}}_{k}-f_{k}\right)}^{2}}.$$
3. R-square:
  (18) $$R^{2}=1-\frac{\sum_{k=1}^{K}{\left({\hat{f}}_{k}-f_{k}\right)}^{2}}{\sum_{k=1}^{K}{\left({\hat{f}}_{k}-{\bar{f}}_{k}\right)}^{2}}\;,\;{\bar{f}}_{k}=\frac{1}{K}\sum_{k=1}^{K}{\hat{f}}_{k}.$$
4. Held-out NLL: In addition to the three grid-based goodness-of-fit metrics, an out-of-sample probabilistic metric is introduced to reduce potential bias toward empirical-density reconstruction. For each scenario, the available samples are randomly divided into a fitting subset and a held-out subset. After a model is fitted on the fitting subset, its test NLL on the held-out samples ${\{x}_{i}^{test}{\}}_{i=1}^{N_{test}}$ is computed as

(19) $$NLL=-\frac{1}{N_{test}}\sum_{i=1}^{N_{test}}\log f(x_{i}^{test}).$$

A smaller NLL indicates that the model assigns higher probability density to unseen samples and therefore has better probabilistic generalization. Unlike SSE, RMSE, and R-square, this metric is evaluated directly on raw held-out observations rather than on the empirical PDF grid and is thus used as a complementary out-of-sample validation rather than a replacement for the original fitting metrics [39].

For each vehicle, each channel, and each angular domain (full-azimuth or directional-window), the URCS-SDE is considered to achieve optimal fitting performance only if it simultaneously yields a lower SSE, RMSE and held-out NLL and a higher R-square than all other models. This criterion is intentionally stringent, ensuring that the superiority of the proposed model is not reflected in a single metric but rather in a consistent and comprehensive improvement in overall fitting quality.

### 3.3. Experimental Workflow and Reproducibility

The experimental workflow in this study is designed to be fully automated, cross-scenario-consistent, and reproducible. This ensures that performance differences across vehicles, channels, and angular domains are solely attributed to model expressiveness, rather than inconsistencies in preprocessing, discretization, or evaluation protocols. For each vehicle, the six channels (20 HH, 20 VV, 40 HH, 40 VV, 60 HH, and 60 VV) are processed independently. For each channel, one full-azimuth scenario and four directional-window scenarios (0° ± 5°, 90° ± 5°, 180° ± 5°, and 270° ± 5°) are evaluated. Each scenario produces the KDE-based empirical reference density and the fitted densities from the URCS-SDE and the five classical models, as well as three objective evaluation metrics computed on a common histogram grid. All results are exported and stored for further analysis. The detailed implementation steps are summarized as follows:

1. Data loading and cleaning.

The azimuth angle φ and channel amplitude $\sigma_{c}\left(\varphi \right)$ are first extracted from the raw files. Non-numeric entries are treated as missing values and removed. Samples are sorted by azimuth angle to ensure that window extraction remains continuous and reliable across the circular boundary at 0°/360°. At this stage, only minimally necessary preprocessing is performed, avoiding any artificial alterations that could bias the distributional shape. And no additional normalization or transformation is applied, except those required for numerical feasibility in parametric fitting.

2. Angular-domain definition and sample extraction.

In the full-azimuth scenario, all samples over 0–360° are aggregated, yielding the set $X_{full}={\left\{x_{i}\right\}}_{i=1}^{N}$. For each directional window, samples within ±5° are extracted using the circular mapping operator wrap(·), producing $X_{\varphi_{0}}={\left\{x_{i}\right\}}_{i=1}^{N_{\varphi_{0}}}$. To avoid unstable estimation in overly small directional windows, a fixed minimum sample threshold of $N_{min}=40$ is imposed. For any vehicle–channel–window combination with $N_{v,c,\varphi_{0}}<40$, the scenario is excluded from quantitative comparison and recorded as a skipped case in the scenario-accounting summary.

3. Empirical reference density construction.

For each scenario, a uniform amplitude grid ${\left\{x_{j}\right\}}_{j=1}^{M}$ is constructed over the sample range, with moderate boundary margins to prevent truncation in visualization. Gaussian-kernel KDE is then applied on this grid to obtain ${\hat{f}}_{KDE}\left(x_{j}\right)$, which serves as the empirical reference density.

4. Model fitting and regularization.

For each scenario, both the five classical parametric models (via MLE) and the URCS-SDE are fitted simultaneously. To ensure numerical stability and physical plausibility, all models follow consistent regularization rules: (1) if a classical model degenerates or fails, a stable fallback Gaussian model via variance matching is used; (2) all PDFs are evaluated on the same amplitude grid as KDE; (3) smoothing and shape constraints are applied to suppress spurious oscillations; (4) each final density is normalized. The URCS-SDE further integrates the three basis functions through weighted least-squares calibration on histogram centers, yielding a smooth unimodal PDF highly aligned with the empirical reference density.

5. Metric calculation and optimal judgment.

To guarantee fair comparability, quantitative metrics are not computed directly on the KDE grid but on a unified set of histogram bin centers. Specifically, using adaptive binning based on the Freedman–Diaconis rule produces bin centers ${\left\{C_{k}\right\}}_{k=1}^{K}$ and empirical densities $\left\{{\hat{f}}_{k}\right\}$. All model outputs are interpolated onto the same $\left\{C_{k}\right\}$ for SSE, RMSE, and R-square computation. In addition, for the held-out NLL metric, the same scenario samples are partitioned into fitting and test subsets using a fixed random seed, so that the probabilistic comparison remains reproducible and strictly consistent across all competing models. The strict optimality criterion in Section 3.2 is then applied to determine whether the URCS-SDE achieves optimal performance in all scenarios.

6. Result output and reproducibility control.

For each scenario, the corresponding figure and table data are exported and stored for statistical aggregation and verification. Given identical input data and software library versions, the entire workflow is deterministic, enabling direct reproduction of all experimental figures and conclusions. In addition, the Supplementary Materials provide scenario-accounting table reports $N_{v,c,\varphi_{0}}$ for every vehicle–channel–window combination, together with the retained or skipped flags; the overall skipped proportion is reported over all 48 directional-window scenarios considered in this work.

## 4. Results and Discussion

### 4.1. Visual Comparison of PDF Fits

**Figure 2:**
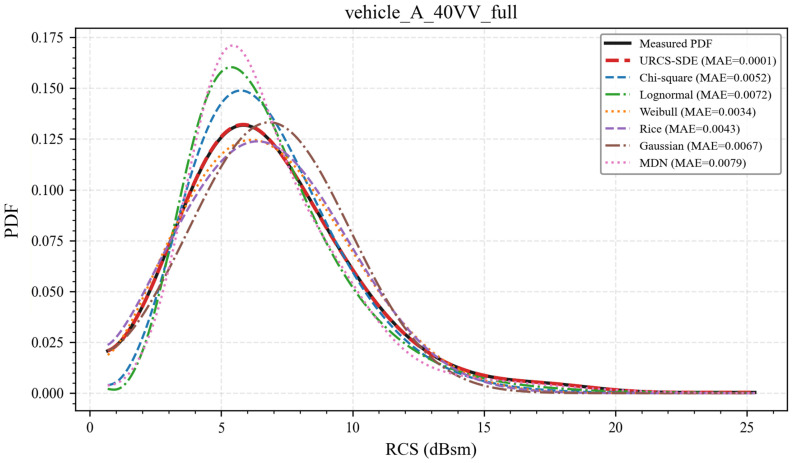
Comparison of PDF and different distribution model fitting PDF curves for the RCS of a target with a 40-degree depression angle and VV polarization under full-azimuth conditions. All measured RCS samples from the specified vehicle A and channel are used.

**Figure 3:**
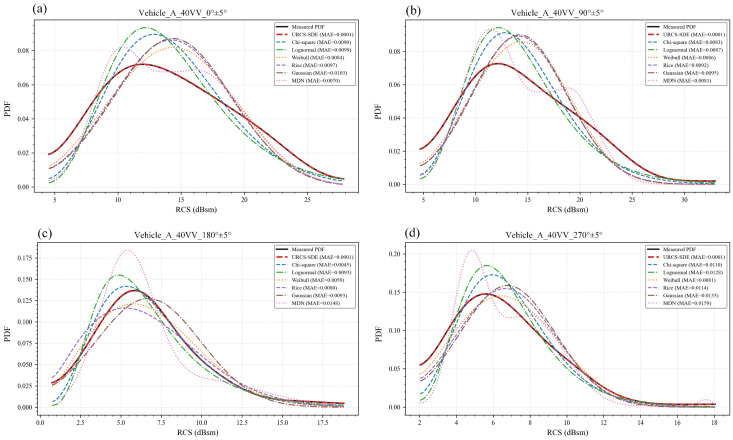
Comparison of PDF and PDF curves fitted by different distribution models for the RCS of a target with a 40-degree depression angle, VV polarization, and four major directional windows. For each subplot, samples are selected from the specified vehicle A and channel within the corresponding azimuth window: (**a**) 0° ± 5° direction, (**b**) 90° ± 5° direction, (**c**) 180° ± 5° direction, and (**d**) 270° ± 5° direction.

To provide a representative qualitative analysis, the 40 VV configuration of vehicle A is taken as an illustrative example. Figure 2 and Figure 3 show the PDF fitting results for the full-azimuth case and the four principal directional windows, respectively. In each plot, the black solid curve denotes the KDE-smoothed empirical PDF constructed from the measured RCS samples, while the fitted curves from the five classical parametric models, the regularized MDN baseline, and the URCS-SDE are shown using different colors and line styles. The legend also includes the mean absolute error (MAE) between each model’s PDF and the experimental one, allowing for both qualitative and simple quantitative comparisons within the same figure. Its purpose is to provide a quick single-number indication of curve deviation in multi-model plots, whereas the formal quantitative comparison and all subsequent conclusions are based on SSE, RMSE, R-square and held-out NLL reported in the tables.

As shown in Figure 2, under full-azimuth conditions, the URCS-SDE fitted curve almost perfectly coincides with the experimental PDF and accurately captures the peak location, overall shape, and tail decay, with its MAE reduced to the 10^−4^ order of magnitude, significantly lower than that of the other models. In contrast, while the classical parametric models and MDN retain unimodal and smooth characteristics, they often exhibit peak shifts (with the main peak either slightly to the left or right) or shape mismatches (such as systematic underestimation or overestimation of the density for rare high-RCS events). Therefore, these models typically struggle to strike a balance between peak height, skewness, and tail behavior, and such deficiencies are reflected in both their visual discrepancies and larger MAE values.

As shown in Figure 3, in the directional-window scenario, the advantage of the URCS-SDE becomes even more pronounced. Due to the contraction of the angular domain, the PDF exhibits stronger directional dependence. Even after smoothing, classical models and the MDN often fail to match the shape near the main peak or in the tail region. In contrast, the URCS-SDE consistently maintains a high degree of agreement with the experimental PDF across all four major azimuths, with accurate peak alignment and overall curve shape consistency. Its MAE remains at an extremely low level. This demonstrates that the URCS-SDE does not merely smooth the empirical curve but is capable of stably reconstructing the true scattering statistical properties in narrow angular domains.

In summary, the results in Figure 2 and Figure 3 validate the ability of the URCS-SDE to characterize the RCS PDF under the 40 VV configuration from both the visual shape and MAE perspectives. This advantage remains consistent across both the full-azimuth and directional-window scenarios. Similar results and conclusions were obtained for other elevation polarizations and vehicles tested.

### 4.2. Quantitative Comparison and Overall Results

To corroborate the visual comparisons in Figure 2 and Figure 3, this section quantitatively evaluates the representative 40 VV case using four formal metrics: SSE, RMSE, R-square and held-out NLL. The first three metrics are computed on the same histogram bin centers and therefore measure how accurately each model reconstructs the empirical-density shape under a unified grid-based protocol. Unlike them, NLL is evaluated directly on unseen samples and therefore serves as a complementary indicator of probabilistic generalization rather than curve reconstruction accuracy alone. Two summary tables are provided: Table 1 corresponds to the full-azimuth scenario, while Table 2 corresponds to the four directional-window scenarios. The corresponding all-condition results for both vehicle A and vehicle B are provided in Tables S3 and S4 in the Supplementary Materials.

As shown in Table 1, the URCS-SDE achieves the best values across the three empirical-density reconstruction metrics and is further supported by the held-out NLL result. This indicates that the “near-perfect overlap” between the URCS-SDE and the experimental PDF in Figure 2 is not merely a visual impression but is quantitatively reflected in substantially reduced errors and an almost complete explanation of the variance in the empirical distribution. In contrast, although the classical parametric models and MDN can also generate smooth unimodal curves, their errors are typically caused by systematic mismatches, most notably peak-location or peak-magnitude biases and inconsistent tail thickness, resulting in a larger SSE/RMSE/held-out NLL and smaller R-square.

Table 2 further examines model robustness under the directional-window settings. These cases are more challenging due to reduced sample sizes and stronger directional scattering characteristics. Nevertheless, the URCS-SDE consistently maintains a clear advantage across all four major azimuths, achieving the best SSE, RMSE, R-square and held-out NLL values and significantly outperforming the other models. This result is consistent with Figure 3, demonstrating that the URCS-SDE can stably preserve the main peak structure and overall decay trend without introducing abnormal oscillations. By contrast, the classical models often exhibit uneven performance across directions and can show pronounced mismatches in certain azimuths, indicating that a single parametric family is insufficient to robustly capture direction-dependent skewness and tail behavior. In addition, the regularized MDN baseline remains less stable than the URCS-SDE in peak alignment and tail control under the present one-dimensional limited-sample setting, especially in directional windows.

In summary, the evidence from Table 1 and Table 2 and Figure 2 and Figure 3 is mutually supportive. Under the representative 40 VV configuration, the URCS-SDE not only significantly outperforms traditional parametric models and the MDN in the full-azimuth aggregated statistics but also remains stably superior under azimuth-resolved directional windows, i.e., it consistently achieves the best performance in both full-azimuth and four major directional-window scenarios. The results reported in the Supplementary Materials for other elevation–polarization configurations and vehicle datasets further suggest that this conclusion is broadly applicable.

To further assess stability under reduced window sample sizes, an additional supplementary analysis was performed on all retained directional-window scenarios by repeated random subsampling within each window and re-fitting of all models under the same evaluation protocol. The results show that, although performance variability increases for all methods as the sample size decreases toward the minimum threshold, the URCS-SDE exhibits a comparatively more gradual degradation and preserves its ranking advantage in most retained scenarios. This confirms that the proposed method is not only accurate under the nominal window samples but also relatively stable in the moderate-sample regime relevant to narrow-angle RCS modeling.

### 4.3. Interpretation and Engineering Implications

The advantage of the URCS-SDE can be further understood through the order-level bias–variance analysis in Section 2.2.3. A correctly specified parametric density estimator benefits from the parametric variance rate $O(n^{-1})$, but this advantage is fragile because once the assumed family is misspecified, the risk contains a non-vanishing approximation term $B_{par}^{2}$. By contrast, KDE avoids rigid family misspecification and attains the standard one-dimensional nonparametric risk envelope $O\left(h^{4}\right)+O((nh{)}^{-1})$; however, in narrow directional windows, its reduced effective sample size makes the variance term more dominant, which explains the mild oscillations or weak spurious modes often observed in practice. The URCS-SDE is designed precisely for this intermediate regime: it preserves the data-adaptive flexibility of KDE, while the unimodal projection suppresses variance-driven local irregularities under an approximately unimodal prior, and the ridge-regularized weighted calibration further stabilizes the combination of the three basis components. Therefore, relative to classical parametric models, the URCS-SDE reduces approximation bias for skewed and heavy-tailed narrow-angle RCS densities; relative to raw KDE, it lowers effective finite-sample variance without discarding the nonparametric adaptability of the pilot estimate. This interpretation also clarifies the finite-sample boundary of applicability. When the misspecification bias of a rigid parametric family exceeds the residual structured bias introduced by the weak unimodal/tail regularization, a semiparametric estimator becomes preferable before the large-sample regime of raw KDE is fully reached. This is exactly the operating region considered in this work: bounded, skewed, and approximately unimodal narrow-angle RCS densities with limited directional-window samples. In such cases, the URCS-SDE provides a more favorable practical balance between model bias and estimator variance than either of the two extremes taken separately.

It should also be emphasized that unimodality is adopted here as a weak structural prior for the typical narrow-angle scenarios considered in this study, rather than as a universal physical law for all targets or all aspect windows; when persistent physically meaningful multimodality is present, the same framework can in principle be extended by replacing the unimodal projection with a multi-peak shape constraint.

From an engineering perspective, the semiparametric PDFs produced by the URCS-SDE provide a compact and directly actionable statistical representation of ground-vehicle scattering characteristics. First, they enable the construction of probabilistic RCS libraries indexed by elevation angle and polarization. For each polarization and angular-domain combination, a smooth density function rather than a single mean value is used, allowing system-level Monte Carlo simulations to more realistically reflect RCS fluctuations and thereby improve the credibility of performance evaluations. Second, accurate tail modeling is critical for practical performance metrics. Rare high-RCS events can dominate long-range detection, whereas low-RCS events are often responsible for missed detections and track interruptions. The tail regularization and weighted calibration in the URCS-SDE enhance the accuracy of low-probability regions, making it particularly suitable for robustness analysis and sensitivity assessment. Moreover, the cross-channel and cross-angular-domain differences captured by the URCS-SDE can serve as statistical “fingerprints” for vehicle recognition and multi-sensor fusion. Differences between HH/VV polarizations and between directional windows—in terms of peak location, distribution spread, and tail probability—can be exploited as discriminative cues for classification and fusion, as distributional shapes often encode richer information than scalar features.

Consequently, the URCS-SDE provides an accurate, stable, and physically meaningful probabilistic characterization of ground-vehicle RCS fluctuations across different elevation configurations, co-polarization modes, and azimuthal domains. This, in turn, offers a reliable and consistent statistical foundation for downstream tasks such as detection performance evaluation, statistical simulation, target feature analysis, and system design.

### 4.4. Simple Downstream Application: Threshold-Based Detection-Support Estimation

To provide a direct engineering-oriented validation, we further examine a simple downstream quantity derived from the fitted RCS PDFs, namely the threshold-exceedance probability. For a threshold τ, the model-implied exceedance probability is defined as

(20) $$P_{ex}\left(\tau \right)=P\left(\sigma >\tau \right)=\int_{\tau }^{\infty }f_{\sigma }(x)dx.$$

Under fixed receiver settings and background assumptions, this quantity can be interpreted as a simple detection-support indicator: a larger exceedance probability at a given threshold implies that the target more frequently produces returns above the decision level.

**Figure 4:**
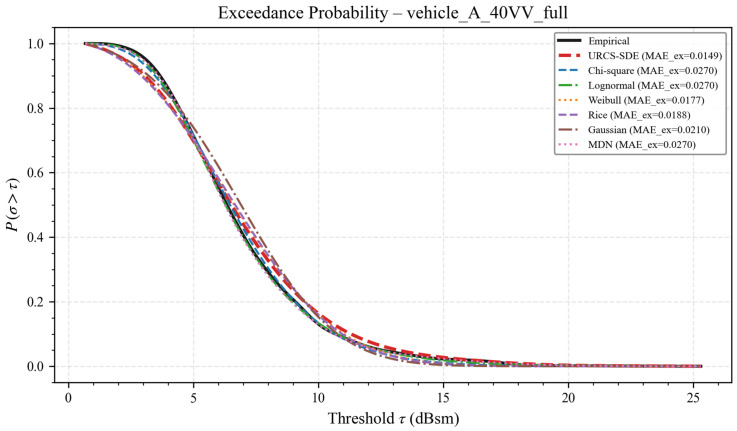
Threshold-exceedance probability curves for vehicle A under the 40 VV channel and full azimuth.

**Figure 5:**
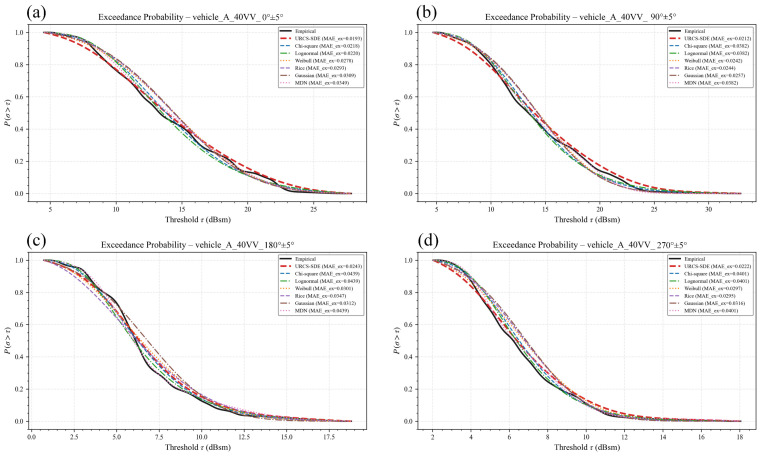
Threshold-exceedance probability curves for vehicle A under the 40 VV channel and the four principal directional windows.

Figure 4 and Figure 5 respectively report representative results for vehicle A under the 40 VV configuration, including the full-azimuth case and the four principal directional windows. In each panel, the empirical exceedance curve computed directly from the measured samples is used as the reference, and the model-implied exceedance curves are evaluated on the same threshold grid. The result shows that the exceedance-probability curve derived from the URCS-SDE tracks the empirical reference more closely over the practically relevant threshold range than the classical baselines and MDN. Moreover, the URCS-SDE has significantly lower errors. This indicates that the advantage of the URCS-SDE is not limited to density curve fitting itself but also carries over to a downstream threshold-based quantity that is directly relevant to target detectability assessment. Therefore, the proposed model has great potential for application in other related engineering projects.

## 5. Conclusions

This paper addresses the problem of statistical characterization of ground-vehicle RCS fluctuations under multiple elevation configurations and HH/VV co-polarization channels, with modeling and evaluation conducted for both full-azimuth and four directional-window scenarios. To overcome the limited expressiveness and fitting instability of classical single-distribution models, a novel model called a URCS-SDE is proposed. The URCS-SDE combines KDE to capture data-adaptive shapes, unimodal shape projection to impose structural regularization, a beta-type tail template to constrain boundary decay, and weighted least-squares calibration for coefficient estimation, followed by smoothing and normalization to yield a stable and physically meaningful unimodal PDF. In systematic experiments involving two vehicle types, six channels, and multiple angular-domain scenarios, the URCS-SDE consistently outperforms five classical parametric baselines and a representative regularized MDN baseline under a unified evaluation protocol. And under a stringent criterion requiring simultaneous minimization of SSE, RMSE and held-out NLL and maximization of R-square, the URCS-SDE achieves optimal fitting performance across all evaluated scenarios and exhibits consistent and stable advantages under varying elevation configurations, polarization modes, and angular domains. These quantitative results are mutually corroborated by the peak-alignment accuracy and tail-matching quality observed in the fitted curves, demonstrating that the URCS-SDE can more effectively capture variations in the scattering statistical characteristics of typical ground vehicles under diverse conditions. In addition, the threshold-based detection-support example demonstrates that the benefit of the URCS-SDE can transfer from density reconstruction to a directly engineering-relevant downstream quantity. Therefore, the URCS-SDE offers an accurate, stable, and practically reproducible probabilistic characterization framework for ground-vehicle RCSs and can be used to complete many downstream tasks in engineering.

Future work will extend the URCS-SDE to multivariate modeling scenarios (e.g., joint frequency–azimuth–polarization RCS distributions) and conduct systematic comparisons and hybrid integrations with rigorously regularized complex deep density models on larger-scale datasets, to further explore pathways toward enhanced expressive power while preserving engineering interpretability.

## Figures and Tables

**Table 1:** Quantitative comparison for the 40 VV full-azimuth case using SSE, RMSE, R-square, and held-out NLL, where the first three metrics evaluate empirical-density reconstruction and NLL evaluates out-of-sample probabilistic generalization.

Model	SSE	RMSE	R-Square	Held-Out NLL
Chi-square	0.017678	0.007676	0.97109	3.3090
Lognormal	0.040562	0.011628	0.933666	3.3090
Weibull	0.00567	0.004347	0.990728	2.5388
Rice	0.009171	0.005529	0.985002	2.5514
Gaussian	0.026706	0.009435	0.956326	2.5687
MDN	0.052341	0.013209	0.914404	3.3090
URCS-SDE	0.000005	0.000127	0.999992	2.3069

**Table 2:** Quantitative comparison for the 40 VV directional-window cases using SSE, RMSE, R-square, and held-out NLL, where the first three metrics quantify grid-based empirical-density agreement and NLL provides complementary held-out probabilistic validation.

Azimuth	Model	SSE	RMSE	R-Square	Held-Out NLL
0° ± 5°	Chi-square	0.016197	0.007348	0.973747	3.0509
	Lognormal	0.058232	0.013932	0.905615	3.0509
	Weibull	0.015013	0.007074	0.975666	2.3133
	Rice	0.032670	0.010435	0.947048	2.3602
	Gaussian	0.040480	0.011616	0.934388	3.0509
	MDN	0.123663	0.020303	0.799562	2.3455
	URCS-SDE	0.000007	0.000157	0.999988	2.1084
90° ± 5°	Chi-square	0.033064	0.010498	0.760987	3.0020
	Lognormal	0.041598	0.011775	0.699302	2.9853
	Weibull	0.027495	0.009573	0.801247	3.0248
	Rice	0.037521	0.011183	0.728769	3.0433
	Gaussian	0.042102	0.011847	0.695653	3.0513
	MDN	0.021373	0.008441	0.845501	3.8850
	URCS-SDE	0.000001	0.000068	0.999996	2.7511
180° ± 5°	Chi-square	0.069516	0.015222	0.914058	2.3509
	Lognormal	0.104010	0.018620	0.871412	3.0926
	Weibull	0.033063	0.010498	0.959124	2.3781
	Rice	0.065950	0.014827	0.918466	2.4042
	Gaussian	0.093765	0.017679	0.884079	2.4266
	MDN	0.162584	0.023280	0.798997	2.4647
	URCS-SDE	0.000005	0.000132	0.999994	2.1404
270° ± 5°	Chi-square	0.031203	0.010198	0.825835	3.3150
	Lognormal	0.036611	0.011047	0.795649	3.2540
	Weibull	0.032442	0.010399	0.818920	3.5909
	Rice	0.037423	0.011169	0.791113	3.5094
	Gaussian	0.040790	0.011660	0.772322	3.5312
	MDN	0.029391	0.009898	0.835947	3.7649
	URCS-SDE	0.000002	0.000083	0.999988	3.0984

## Data Availability

The original contributions presented in the study are included in the article material; further data that support the findings of this study are available on request from the corresponding author upon reasonable request.

## Supplementary Materials

The following supporting information can be downloaded at: https://www.mdpi.com/article/10.3390/s26092572/s1, Table S1: Default implementation constants, functional roles and sensitivity ranges of URCS-SDE; Table S2: Scenario-accounting table reporting sample size, retained/skipped status, and overall skipped proportion for all directional-window cases; Table S3: Full-azimuth quantitative comparison for vehicle A and vehicle B across all elevation–polarization channels; Table S4: Directional-window quantitative comparison for vehicle A and vehicle B across all elevation–polarization channels.

## Abbreviations

The following abbreviations are used in this manuscript:

RCS	Radar Cross Section
URCS-SDE	Unimodal RCS Semiparametric Density Estimator
KDE	Kernel Density Estimation
PDF	Probability Density Function
AMISE	Asymptotic Mean Integrated Squared Error
MISE	Mean Integrated Squared Error
SSE	Sum of Squared Errors
RMSE	Root Mean Squared Error
R-square	Coefficient of Determination
NLL	Negative Log-Likelihood
MDN	Mixture Density Network
MLE	Maximum Likelihood Estimation
HH	Horizontal–Horizontal
VV	Vertical–Vertical
MAE	Mean Absolute Error
